# SLE Plasma Profiling Identifies Unique Signatures of Lupus Nephritis and Discoid Lupus

**DOI:** 10.1038/s41598-019-50231-y

**Published:** 2019-10-08

**Authors:** Michael A. Smith, Jill Henault, Jodi L. Karnell, Melissa L. Parker, Jeffrey M. Riggs, Dominic Sinibaldi, Devon K. Taylor, Rachel Ettinger, Ethan P. Grant, Miguel A. Sanjuan, Roland Kolbeck, Michelle A. Petri, Kerry A. Casey

**Affiliations:** 1 AstraZeneca, Gaithersburg, MD USA; 2 Johns Hopkins University School of Medicine, Baltimore, MD USA; 3 Present Address: Allen Institute for Immunology, 615 Westlake Ave N, Seattle, WA 98109 USA

**Keywords:** Diagnostic markers, Lupus nephritis, Diagnostic markers, Systemic lupus erythematosus

## Abstract

Systemic lupus erythematosus (SLE) impacts multiple organ systems, although the causes of many individual SLE pathologies are poorly understood. This study was designed to elucidate organ-specific inflammation by identifying proteins that correlate with SLE organ involvement and to evaluate established biomarkers of disease activity across a diverse patient cohort. Plasma proteins and autoantibodies were measured across seven SLE manifestations. Comparative analyses between pathologies and correlation with the SLE Disease Activity Index (SLEDAI) were used to identify proteins associated with organ-specific and composite disease activity. Established biomarkers of composite disease activity, SLE-associated antibodies, type I interferon (IFN), and complement C3, correlated with composite SLEDAI, but did not significantly associate with many individual SLE pathologies. Two clusters of proteins were associated with renal disease in lupus nephritis samples. One cluster included markers of infiltrating leukocytes and the second cluster included markers of tissue remodelling. In patients with discoid lupus, a distinct signature consisting of elevated immunoglobulin A autoantibodies and interleukin-23 was observed. Our findings indicate that proteins from blood samples can be used to identify protein signatures that are distinct from established SLE biomarkers and SLEDAI and could be used to conveniently monitor multiple inflammatory pathways present in different organ systems.

## Introduction

Systemic lupus erythematosus (SLE) is an autoimmune, multisystem disease with a complex pathogenesis. Skin involvement occurs in approximately 70% of patients with SLE, most commonly as erythematous lesions (known as acute cutaneous lupus [ACL]) or in the more localised scarring form, discoid lupus (DL)^1^. Thrombocytopenia occurs in 7–30% of patients with SLE and is significantly associated with increased morbidity^2^. Sjögren’s syndrome (SS), a condition that afflicts the salivary glands and causes hyposalivation, occurs in approximately 10% of patients with SLE^3^. Additionally, about 50% of patients with SLE develop glomerulonephritis^4^. The majority of these individuals develop proliferative nephritis, lupus nephritis (LN) class III and IV, defined by subendothelial immune complex deposition in the glomerulus^5^. Approximately 10–20% of patients with SLE acquire membranous nephritis, also termed LN class V, defined by subepithelial immune complex deposits in the glomerulus^5^. The individual drivers behind development of these prevalent SLE disease manifestations, and the underlying causes of heterogeneity, are not fully understood.

Autoantibodies and type I interferon (IFN) are thought to contribute to the pathogenesis of SLE. Anti–double-stranded DNA (anti-dsDNA), Ro, La, and Sm autoantibodies are prevalent in the circulation of patients with SLE^6^, and immune complex deposits have been found in multiple tissues including the skin, synovium, kidneys, blood vessels, and pleural membranes^7^. Endosomal sensing of these immune complexes by plasmacytoid dendritic cells triggers potent production of type I IFN. Type I IFN–inducible transcripts and chemokines are highly prevalent in the blood and tissues of patients with SLE^8–11^. Indicative of a potential causal role of these components in SLE, serological transfer of SLE autoantibodies in mice or type I IFN administration in humans induces SLE symptoms^12,13^.

Further implicating autoantibodies and type I IFN as drivers of SLE manifestations, belimumab, an antibody that moderately reduces anti-dsDNA autoantibodies, has demonstrated efficacy in the treatment of SLE^14,15^. However, the full spectrum of SLE-related activity is not fully explained using established biomarkers of these pathways. Patients with SLE can present with active disease in the absence of autoantibodies or in the absence of an elevated type I IFN gene signature, suggesting involvement of other pathways^16,17^. Moreover, elevated autoantibodies or an elevated type I IFN gene signature can be found in the blood and tissues of asymptomatic patients^16–18^. These findings suggest additional unknown mechanisms also contribute to SLE pathologies.

To identify molecular signatures of these inflammatory mechanisms, we hypothesised that comparative analysis across an SLE cohort focused on prevalent SLE-related organ manifestations would reveal molecular signatures of SLE-related organ damage, as blood protein composition can reflect pathological changes in tissues. To this end, we utilised a custom panel of inflammation-associated proteins to profile plasma from a cohort consisting of seven of the most prevalent SLE manifestations: DL, ACL, secondary SS, thrombocytopenia, LN III, LN IV, and LN V. First, the relationship was characterised between individual SLE manifestations represented in the cohort and established biomarkers of composite disease activity: SLE-associated autoantibodies, C3, and type I IFN–inducible chemokines. Second, it was investigated whether signatures of organ-specific disease could be identified through comparative analysis across different patient groups. Finally, by assessing the ability of composite disease activity scores to reflect organ-specific signatures, it was determined whether the signatures of organ-specific activities were unique to specific organ systems or systemic in nature.

## Results

### Correlation of established SLE biomarkers with composite disease activity but not specific SLE manifestations

**Figure 1:**
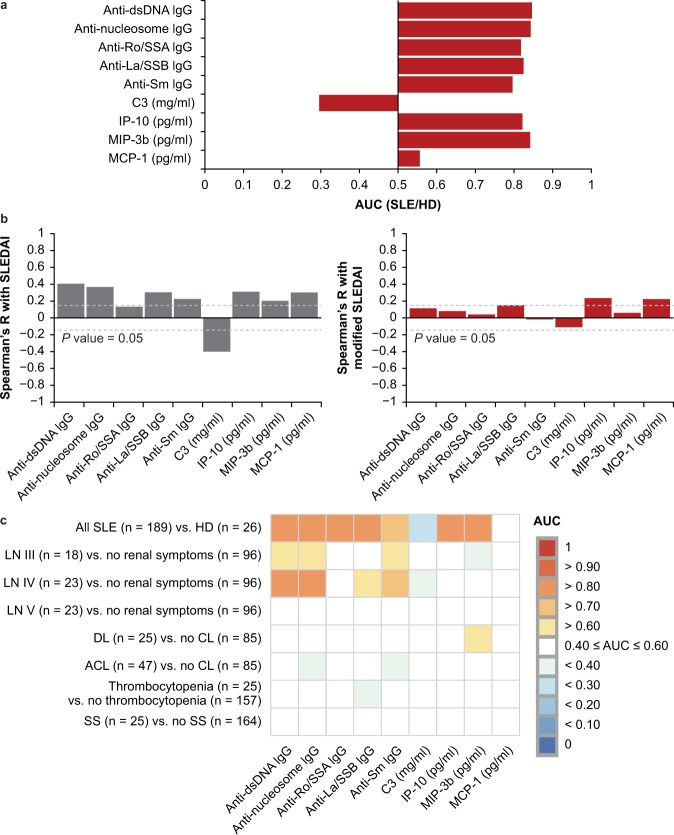
Association between established systemic lupus erythematosus (SLE) biomarkers and different SLE pathologies. (**a**) Area under the curve (AUC) for established SLE biomarkers in all SLE (n = 189) versus healthy donors (HD; n = 26). (**b**) Spearman’s rank correlation of established SLE biomarkers with SLE Disease Activity Index (SLEDAI) and modified SLEDAI to exclude serological components, C3, and anti–double-stranded DNA (anti-dsDNA) antibody components of score (modified SLEDAI). Dotted lines represent Spearman’s R critical values needed to achieve statistical significance with α = 0.05. (**c**) AUC of established biomarkers compared between plasma samples drawn from patients with SLE with different manifestations. ACL = acute cutaneous lupus; CL = cutaneous lupus; DL = discoid lupus; Ig = immunoglobulin; LN = lupus nephritis; SS = Sjögren’s syndrome.

To measure the association between established SLE biomarkers and disease activity across the diverse SLE cohort, we first compared the prevalence of five autoantibodies (anti-dsDNA immunoglobulin [Ig] G, anti-nucleosome IgG, anti-Ro IgG, anti-La IgG, and anti-Sm IgG) used to diagnose and monitor SLE disease activity^6,19,20^, along with three IFN-inducible chemokines (IP-10, MCP-1, MIP-3β)^11^ and C3 across the combined cohort and the seven defined SLE manifestations. Demographic and clinical characteristics of the cohort are described in Table 1. There was a high prevalence of anti-dsDNA, anti-nucleosome, anti-Ro, anti-La, and anti-Sm IgG autoantibodies across the entire SLE cohort (area under the curve [AUC] > 0.75, *P* < 0.001) and within each manifestation (AUC > 0.70, *P* < 0.05) compared with healthy donors. Similarly, IP-10 and MIP-3β were elevated across the cohort (AUC > 0.75, *P* < 0.001) and within each manifestation (AUC > 0.70, *P* < 0.05). Low levels of C3 were also observed across the SLE cohort (AUC = 0.3, *P* < 0.001) and within each manifestation (AUC < 0.4, *P* > 0.05). MCP-1 was not significantly elevated in the SLE cohort versus healthy donors (*P* > 0.05) (Fig. 1a). Together, these results suggest that established SLE-associated autoantibodies, type I IFN–inducible chemokines, and low levels of C3 are ubiquitous features of SLE across patients with varied organ-related manifestations.

**Table 1:** Demographics and clinical characteristics.

Parameter	HD (n = 26)	All SLE (n = 189)	Discoid (n = 25)	LN III (n = 18)	LN IV (n = 23)	LN V (n = 23)	Thrombo- cytopenia (n = 25)	ACL (n = 50)	Sjögren’s (n = 25)
Median age	40 (30, 50)	38 (30, 44)	38 (33, 48)	35 (30, 42)	31 (26, 40)	37 (27, 42)	35 (29, 39)	39 (32, 47)	40 (35,49)
Female (%)	46	90	92	94	96	87	92	82	96
Black (%)	12.5	54	80	50	52	61	56	44	48
White (%)	75	40	16	39	35	35	44	48	52
Asian (%)	12.5	3	4	11	4	0	0	4	0
Other race (%)	0	3	0	0	9	4	0	4	0
Median SLEDAI (1Q, 3Q)	—	4 (2, 6)	4 (2, 7)	5 (2, 8)	4 (2, 6)	2 (0, 4)	4 (2, 5)	4 (2, 6)	4 (2,5)
Median LAI (1Q, 3Q)	—	1 (0, 2)	1 (0, 2)	0 (0, 2)	1 (0, 2)	1 (0, 2)	0 (0, 1)	1 (1, 2)	0 (0,1)
Low complement (%)	—	45	56	50	61	43	48	32	40
Increased DNA binding (%)	—	41	40	56	70	26	43	30	36
Median C3 mg/dL (1Q, 3Q)	—	90 (73, 113)	87 (73, 109)	88.5 (73.5, 115.5)	78 (66.5, 89.5)	95 (77.5, 101.5)	82 (67.5, 111)	97.5 (73, 116.25)	108.5 (90, 138.5)
Median C4 mg/dL (1Q, 3Q)	—	16 (11, 24.25)	18 (13, 24)	14.5 (12.25, 24.75)	13 (8.5, 20)	15 (10, 25.5)	16 (11, 25.5)	18 (13, 24.25)	17 (11.75, 23.5)
Key medications									
Prednisone (%)	—	71	88	78	61	78	83	60	64
Hydroxychloroquine (%)	—	57	56	67	61	61	17	72	48
Mycophenolate (%)	—	14	8	28	26	35	0	12	0
Cyclophosphamide (%)	—	3	0	0	0	9	0	8	0
Azathioprine (%)	—	8	12	17	13	13	0	8	0
Methotrexate (%)	—	1	4	0	0	0	0	0	0
Tacrolimus (%)	—	1	0	6	0	0	0	0	0

SLEDAI, Systemic Lupus Erythematosus Disease Activity Index; LAI, Lupus Activity Index; HD, healthy donors; LN, lupus nephritis; ACL, acute cutaneous lupus; 1Q, first quartile; 3Q, third quartile.

Spearman’s rank correlation was used to test for association between each marker and SLE Disease Activity Index (SLEDAI), a measure of composite disease activity. A modified SLEDAI, which lacks the serological components of the score, DNA binding, and hypocomplementaemia, was used to obtain measures of association with organ-specific disease activity^21,22^. All markers examined, except anti-Ro IgG, displayed significant correlation with SLEDAI. Only IP-10 (Spearman’s R = 0.23, *P* = 0.0017) and MCP-1 (Spearman’s R = 0.23, *P* = 0.0017) displayed statistically significant correlations with the modified SLEDAI (Spearman’s R = 0.16, *P* = 0.04) (Fig. 1b). These data suggest that although type I IFN and SLE-associated autoantibodies are pervasive across physically diverse patients and correlate with SLEDAI, these biomarkers do not strongly reflect organ-specific disease activity.

To determine whether type I IFN and autoantibodies could be implicated in disease activity within specific organ systems, we examined the association between SLE-associated autoantibodies, IFN-inducible chemokines, and C3 as well as the presence of individual SLE pathologies. Each biomarker was examined in patients positive for each manifestation and compared with patients negative for the manifestation as reported by SLEDAI and Lupus Activity Index (LAI) components. Aside from the known association between anti-nucleosome autoantibodies and proliferative nephritis, no overt relationship was found between these biomarkers and the manifestations examined (Fig. 1c). Whereas an association between anti-nucleosome IgG and proliferative nephritis has been previously reported^23,24^, we extend this observation and demonstrate associations between proliferative nephritis and all isotypes of anti-nucleosome antibodies, with IgG and IgA being the most prevalent isotypes (Supplemental Table 1). Together, these results support the involvement of anti-nucleosome and related antibodies in the development of proliferative nephritis. These results also suggest that established SLE biomarkers do not distinguish between patients with different organ involvement or manifestations.

### Identification of novel protein signatures of LN

**Figure 2:**
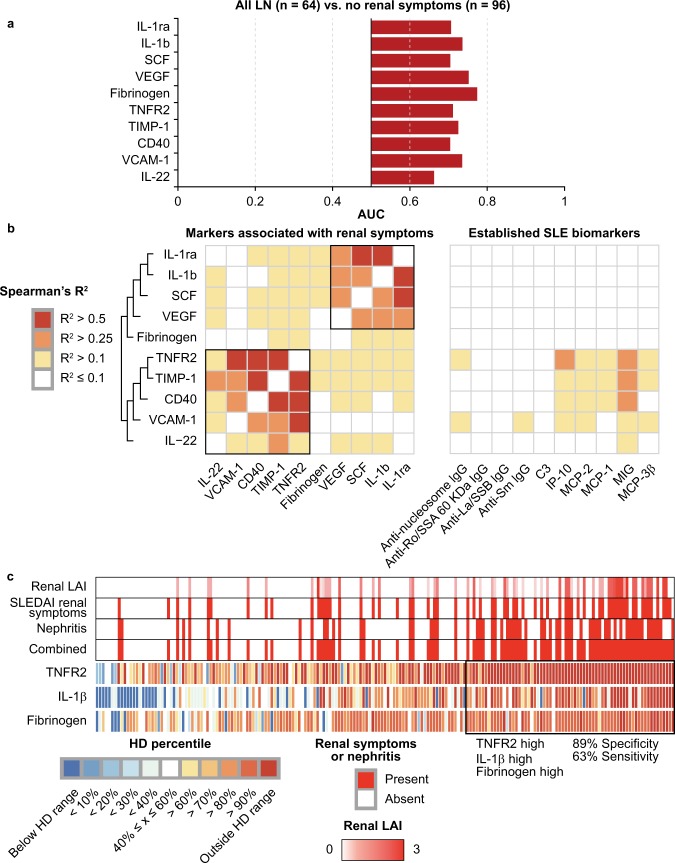
Protein signatures associated with lupus nephritis (LN). (**a**) Area under the curve (AUC) of biomarkers between patients with LN and patients with systemic lupus erythematosus (SLE) without renal activity. (**b**) Correlation matrices displaying correlations in all patients with SLE between markers associated with LN and across traditional SLE biomarkers measured in SLE. Data clustered using 1-(Spearman’s R)^2^ as distance measure and complete linkage as clustering method. (**c**) Heatmap displaying patterns of prevalence of tumour necrosis factor receptor 2 (TNFR2), interleukin (IL)-1β, and fibrinogen across all patients with SLE. Samples ordered by weight sum of TNFR2, IL-1β, and fibrinogen. Heatmap colours represent measurements from SLE samples that were standardised to healthy donor (HD) measurements for each respective biomarker. Red colour bars (top) represent the presence or absence of different renal pathologies or severity of renal Lupus Activity Index (LAI) score. SLEDAI = SLE Disease Activity Index.

As established SLE biomarkers did not strongly distinguish between SLE-related, organ-specific activity, we next investigated whether other proteins differentiated individual SLE pathologies. To this end, we sought to identify proteins associated with LN by comparing analyte prevalence between patients with LN III, IV, and V with a group of patients with SLE who were inactive for renal LAI and SLEDAI components. We measured 387 unique SLE- and inflammatory pathway–associated proteins via protein array, custom Luminex, or enzyme-linked immunosorbent assay (ELISA), including IgG, IgA, and IgM antibody isotypes with 94 individual antigen specificities (Supplemental Table 2). Of these analytes, ten proteins were elevated in the combined group of patients with LN versus patients without renal activity (false discovery rates [FDR] < 0.10) and in each LN class (*P* < 0.05) (Fig. 2a, Supplemental Table 3). All 10 proteins displayed a significant correlation with renal components of the LAI and SLEDAI across the cohort (*P* < 0.05) (Supplemental Table 4). These proteins were consistently elevated in both proliferative and membranous LN samples and associated with renal disease activity.

To test whether these proteins share a common driver or reflect distinct modes of renal inflammation in LN, the correlations between the 10 proteins within the SLE cohort were examined. Spearman’s rank correlations were calculated between each protein and hierarchically clustered using 1-Spearman’s R^2^ as a distance metric between analytes. Two separate clusters were identified, each sharing a Spearman’s R^2^ of at least 0.10 (*P* < 0.001). Cluster 1 contained markers of infiltrating leukocytes (tumour necrosis factor receptor 2 [TNFR2], tissue inhibitor of metalloproteinase-1 [TIMP-1], CD40, IL-22) and leukocyte migration (vascular cell adhesion protein 1 [VCAM-1]). Analytes in this cluster displayed significant (Spearman’s R^2^ > 0.1, *P* < 0.001) correlations with IFN-inducible chemokines and, in some cases, SLE-associated antibodies. Cluster 2 contained possible markers of tissue remodelling, IL-1b, IL-1ra, stem cell factor (SCF), and vascular endothelial growth factor (VEGF). These proteins did not significantly correlate with established SLE biomarkers (Spearman’s R^2^ < 0.1) (Fig. 2b). Forward selection from logistic regression identified IL-1b, TNFR2, and fibrinogen as representative markers. Significance tests on the regression coefficients revealed each of these proteins independently associated with renal activity (*P* < 0.05) (Supplemental Table 5). At the patient level, the three biomarkers distinguished patients with renal activity from patients without renal activity with a mean 89% specificity and 63% sensitivity after 10 iterations of 5-fold cross validation (Fig. 2c). Collectively, these results suggest two signatures that reflect distinct pathways in LN, one of which displays independence to established SLE biomarkers.

**Figure 3:**
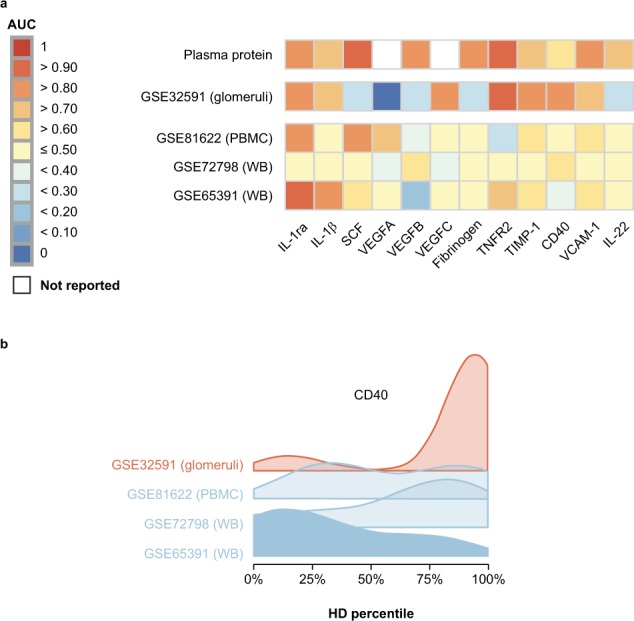
Expression of proteins associated with renal disease in lupus nephritis (LN) blood and glomeruli. (**a**) Area under the curve (AUC) of gene expression between LN and healthy donor (HD) whole blood (WB) from GEO series matrices of GSE65391 and GSE72798; peripheral blood mononuclear cells (PBMCs) from GSE81622; and glomeruli from GSE32591. (**b**) Density plot displaying CD40 expression, representative of several transcripts, in systemic lupus erythematosus (SLE)-afflicted glomeruli and both WB and PBMCs normalised by HD percentile. IL = interleukin.

A key hurdle in advancing the understanding of organ-specific activity is finding blood biomarkers that reflect biological changes in affected tissues. Transcript prevalence was examined for each protein by merging publicly available gene expression profiles from laser-captured glomeruli, whole blood, and peripheral blood mononuclear cell (PBMC) samples to determine whether the cellular sources of these signature proteins can be most readily found in the blood or kidney^25–28^. mRNA transcripts from 7/10 LN signature proteins were found with increased expression in LN-afflicted glomeruli versus glomeruli collected from healthy donors (AUC > 0.70, *P* < 0.05) (Fig. 3a). In contrast, many of the same transcripts were not elevated in LN blood-derived gene expression profiles versus healthy donors. TNFR2, TIMP-1, CD40, and VCAM-1 each displayed elevated expression in the glomeruli (TNFR2, TIMP-1, CD40 AUC > 0.8, *P* < 0.001; VCAM-1 AUC > 0.7, *P* < 0.05) but were not significantly elevated in whole blood gene expression profiles (TNFR2, TIMP-1, CD40, VCAM-1 AUC < 0.7) (a representative density plot of CD40 transcript levels in glomeruli and blood is shown in Fig. 3b). These results are consistent with potential kidney-resident cellular sources of these LN signatures.

### Unique signature differentiates patients with DL

**Figure 4:**
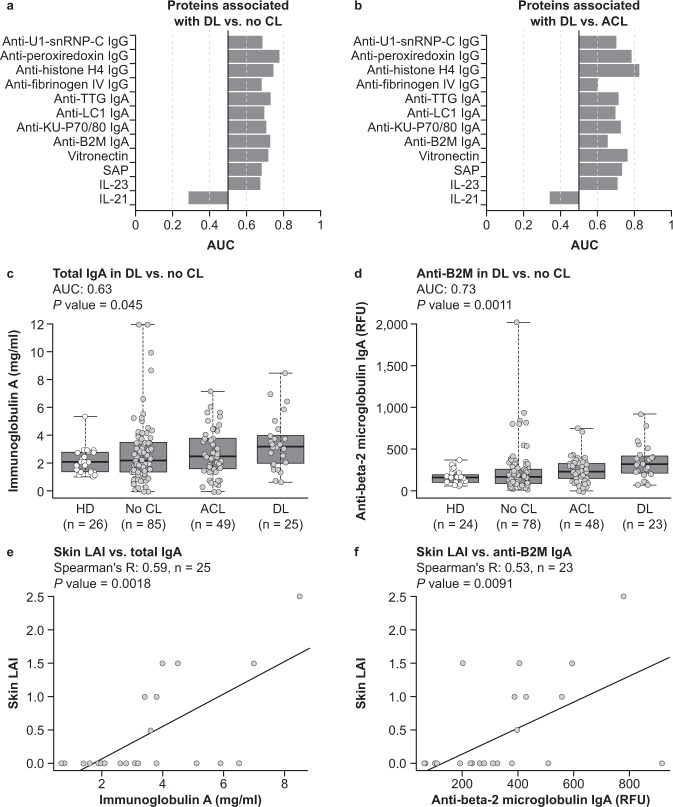
Protein signature associated with discoid lupus (DL). (**a**) Area under the curve (AUC) of biomarkers compared between plasma samples drawn from patients with DL versus those without cutaneous symptoms. (**b**) AUC of biomarkers compared between plasma samples drawn from patients with DL versus those with acute cutaneous lupus (ACL). (**c**,**d**) Anti-β_2_ microglobulin (β2 M) antibodies as measured by antigen microarray and total IgA concentration measured by Rules-Based Medicine in plasma samples from healthy donors (HD), patients with systemic lupus erythematosus (SLE) without cutaneous symptoms, patients with SLE and ACL, and patients with SLE and DL. Box and whiskers represent quartiles of each group. (**e**,**f**) Correlation between skin component of Lupus Activity Index (LAI) and anti-β2 M immunoglobulin (Ig) A and total IgA. Line represents best fit via robust linear regression. CL = cutaneous lupus; IL = interleukin.

To identify proteins associated with DL, we first compared analyte prevalence between patients with DL and patients with SLE without cutaneous involvement (as measured through SLEDAI and LAI) using the same set of analytes assessed for identification of LN signatures (94 IgG, IgA, and IgM antibodies and 105 additional SLE-associated proteins/analytes; Supplemental Table 2). After multiple testing corrections, four IgG antibodies (anti-U1-snRNP-C, peroxiredoxin, histone H4, and fibrinogen IV), four IgA antibodies (anti-TTG, LC1, KU-P70/80, and β2 M IgA), and four proteins (vitronectin, SAP [serum amyloid P-component], IL-23, and IL-21) were identified that differentiated patients with DL (Supplemental Table 6, Fig. 4a). Furthermore, each identified marker displayed significant specificity for patients with DL versus patients with SLE and ACL (*P* < 0.05), except for anti-fibrinogen intravenous IgG (*P* > 0.05) (Supplemental Table 6, Fig. 4b). Overall, these analyses suggest candidate biomarkers specifically elevated in plasma samples from patients with DL have been identified.

Spearman’s rank correlation was measured between each DL-associated marker and skin component of the LAI in patients with DL to further characterise the association between these proteins and DL disease activity. Total IgA antibodies (measured by Rules-Based Medicine [RBM]) and anti-β2 M IgA antibodies (measured through autoantibody microarrays) correlated with the skin component of the LAI (Spearman’s R > 0.5, *P* < 0.01) (Fig. 4c–f). These findings provide evidence for a unique association between IgA autoantibodies and DL disease activity.

**Figure 5:**
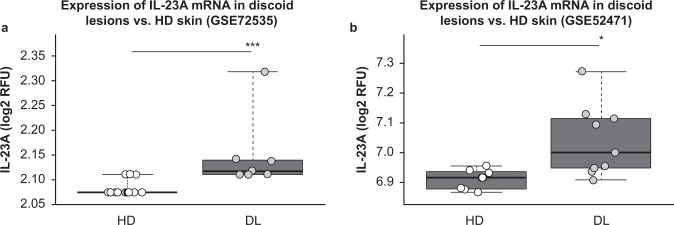
Expression of interleukin (IL)-23 in skin biopsies from patients with discoid lupus (DL) and healthy donors (HD). Gene expression of IL-23A (IL-23) was compared between discoid-afflicted and healthy skin punch biopsies in GEO series matrices (**a**) GSE72535 and (**b**) GSE52471. *P < 0.05; **P < 0.01; ***P < 0.001 (Mann–Whitney U test).

Next, we explored IL-23A expression in gene expression data from two studies that measured relative mRNA expression of discoid lesions versus non-lesional skin from healthy donors to understand the localisation of gene expression of proteins elevated in the plasma of patients with DL. IL-23A expression was elevated in patients with DL in both sets of gene expression profiles^29,30^ (AUC > 0.8, *P* < 0.05), suggesting that discoid lesions could be a source of circulating IL-23 in DL plasma (Fig. 5).

### Signatures reflect local inflammation in kidneys and discoid lesions

**Figure 6:**
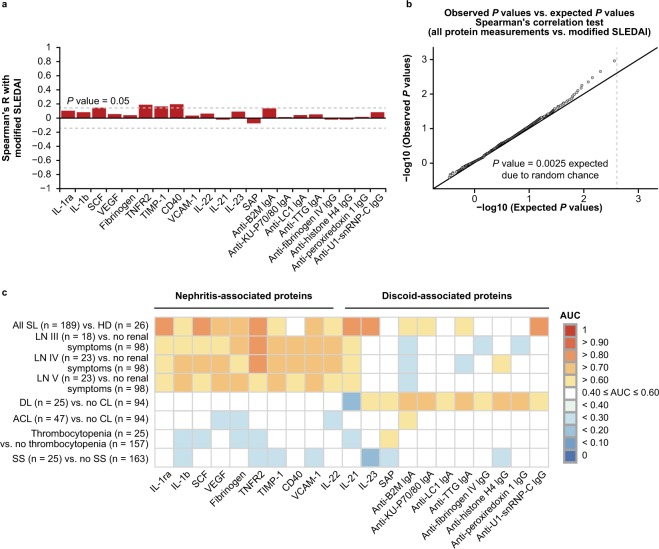
Association between protein signatures, composite disease activity, and individual systemic lupus erythematosus (SLE) pathologies. (**a**) Spearman’s rank correlation of signature proteins with SLE Disease Activity Index (SLEDAI) excluding C3 and DNA binding (modified SLEDAI). Dotted lines represent Spearman’s R critical values needed to achieve statistical significance with α = 0.05. (**b**) Observed *P* values versus expected *P* values from Spearman’s rank correlation test of each analyte versus modified SLEDAI score. Dotted line represents expected minimum *P* value due to random chance. (**c**) Area under the curve (AUC) of antibody and protein measurements associated with lupus nephritis (LN) and discoid lupus (DL) between all patients with SLE and healthy donors (HD) and between patients with SLE presenting with different manifestations. ACL = acute cutaneous lupus; CL = cutaneous lupus; Ig = immunoglobulin; IL = interleukin; SS = Sjögren’s syndrome.

Measurement of disease activity is challenging because of the complex and heterogeneous nature of SLE. Generally, SLE disease activity is quantified using composite indices to consolidate multiple manifestations spanning disparate organ systems into a single score. The SLEDAI composite score consolidates 24 discrete components from 8 organ systems into a single disease activity score and is widely used as a measure of global disease activity in clinical trials and translational research. To determine whether the identified protein signatures were reflective of unique biology observed in LN and DL or indicative of a more global increase in disease activity, we measured the correlation between each protein and composite disease activity as assessed by modified SLEDAI. Individual signature proteins displayed weak correlations with SLEDAI (Spearman’s R < 0.3) (Fig. 6a), suggesting that the signatures we identified do not specify systemic inflammation but rather reflect pathobiology localised to the kidneys and discoid lesions of patients. Collectively, these findings also imply that composite disease activity measures may be insufficient for monitoring local inflammation in these organs.

Because none of these signature proteins sufficiently reflected composite disease activity, we also sought to understand whether any measurements did. To this end, the correlation was measured between each protein and organ-specific disease activity as reported through the modified SLEDAI. No measurements were significantly associated with the modified SLEDAI after performing multiple testing corrections (Fig. 6b). Moreover, expanding the query across all protein measurements failed to reveal any significant correlates of composite disease activity in this cohort, consistent with the notion that organ-associated pathobiology might be highly individualised to the afflicted organ.

Proteins were compared between patients with each pathology with patients in the cohort negative for any symptom of that pathology to further test the hypothesis that these signature proteins are only associated with local inflammation within specific organ systems. There was no significant association of LN or DL protein signatures with other SLE-related manifestations (Fig. 6c), indicating that these signatures are uniquely associated with LN and DL. Therefore, the pathways responsible for these signatures are likely not systemic in nature, but local to the kidney and discoid lesions.

## Discussion

By examining different SLE manifestations in isolation, we have identified protein signatures associated with local inflammation in discoid lesions and lupus glomeruli. Two of the identified signatures also displayed independence to established SLE biomarkers of composite disease activity: SLE-associated autoantibodies, C3, and type I IFN–inducible chemokines. These findings suggest that novel inflammatory pathways contribute to DL and LN in addition to autoantibodies and type I IFN, which are both hypothesised drivers of SLE. Treatment of SLE in the future will need to target different pathways in different patients, based on their organ involvement and the pathways involved.

This study design contrasts to other SLE molecular profiling studies in SLE. The cohort was enriched for key SLE manifestations and was paired with an analysis approach geared towards understanding differences between these subgroups. Even in the cohort enriched for organ involvement, 38% of the SLEDAI score was attributable to the anti-dsDNA and complement components, which are both associated with type I IFN^11,17,22^. After removal of these serological components, no analytes were significantly associated with modified SLEDAI. Rather, proteins were identified that correlated with disease activity within a particular organ. These signatures were not correlated with type I IFN–inducible chemokines. In summary, signatures associated with LN and DL were identified that are not reflected by SLEDAI or modified SLEDAI, providing evidence that uncoupling composite disease activity can reveal unique information distinct from composite disease activity signatures.

The two identified protein signatures that are elevated in LN raise new possibilities. Histological examination of renal biopsies is the gold standard for LN diagnosis and disease monitoring. Pathologists have observed two distinct lesions in these biopsies, termed ‘active’ and ‘chronic.’ Active lesions are characterised by immune complex deposition, leukocyte infiltration, endocapillary hypercellularity, karyorrhexis, fibrinoid necrosis, rupture of the glomerular basement membrane, cellular crescents, and intraluminal immune aggregates^5^. Chronic lesions, however, are composed of glomerular sclerosis, fibrous adhesions, and fibrous crescents^5^. Recent studies have shown that both types of lesions have important associations with kidney function and disease progression in LN. Patients with only active lesions have responded better to conventional immunosuppressive treatment than patients with a mixture of active and chronic lesions^31^. Moreover, patients with a mixture of active and chronic lesions displayed decreased renal survival compared with patients with only active lesions^31^. These findings highlight the importance of identifying circulating measures associated with active inflammation and chronicity to further investigate these axes.

For LN, two independent protein signatures were identified that could reflect the presence of active and chronic lesions in LN kidneys. The first signature, comprised of TNFR2, CD40, TIMP-1, and VCAM-1, may be produced by active lesions. CD40, TNFR2, and TIMP-1 have been shown to be expressed by kidney-infiltrating immune cells in multiple renal diseases. CD40 is expressed by infiltrating mononuclear cells in LN along with two other antibody-mediated renal diseases: IgA nephropathy and anti-neutrophil cytoplasmic antibody (ANCA)–associated vasculitis^32^. TIMP-1 is expressed intraglomerularly by infiltrating neutrophils and macrophages in ANCA-associated vasculitis^33^. TNFR2 increases intrarenally in cases of acute versus chronic renal allograft rejection and is also expressed by infiltrating macrophages and B cells in these biopsies^34^. VCAM-1 is expressed by tubular cells in inflamed kidneys in multiple renal diseases, including LN. VCAM-1 correlates with the number of infiltrating leukocytes and is known to aid in homing of leukocytes to inflamed tissues^35^. Several of these proteins were significantly associated with biomarkers of immune complex deposition, elevated SLE-specific antibodies and decreased C3, further implicating them as a signature of active renal inflammation. Each protein also displayed significant correlation with IFN-inducible chemokines, suggesting these chemokines could be the mediators responsible for attracting immune cells to the site of tissue damage.

The second identified protein signature in LN, comprised of IL-1β, IL-1rA, SCF, and VEGF, could be a signature produced by chronic lesion tissue remodelling in LN glomeruli. *In situ* SCF expression strongly correlates with interstitial fibrosis in patients with various renal diseases including LN^36^. Fibrinogen was also weakly associated with LN in both clusters. Additionally, the other proteins identified in this second cluster seem to be expressed mainly by kidney resident cells, not infiltrating immune cells. IL-1β and IL-1rA are primarily produced by podocytes across multiple renal diseases^37^. VEGF is an angiogenic factor also produced by podocytes and mesangial cells in proliferative nephritis^38^. This second group of proteins correlated with renal disease severity but did not display significant correlation with IFN-inducible chemokines, SLE-associated antibodies, and C3, suggesting that this inflammation is temporally distinct to type I IFN and kidney immune complex deposition. These histological studies provide evidence that these proteins could represent a chronic inflammatory/tissue remodelling axis, with independence to inflammation driven by immune complex deposition or type I IFN production in the kidney. Further work should be performed to understand whether the blood signatures identified correlate with histological measures of active inflammation and chronic tissue remodelling in LN.

Additionally, the finding that multiple IgA antibodies correlate with DL activity raises new hypotheses about the aetiology of DL. IgA is a well-known mediator of skin disease. In linear IgA bullous dermatosis, IgA directed against antigens in the skin are deposited linearly across the basement membrane. Along with recruited neutrophils and plasminogen, these IgA antibodies drive the detachment of the epidermis and the formation of bullae in the skin^39^. In dermatitis herpetiformis, a cutaneous manifestation of celiac disease characterised by pruritic polymorphic lesions, granular deposits of IgA are found in the papillary tips of the dermis along with neutrophil infiltration^40^. For patients with celiac disease, the IgA anti-transglutaminase test helps identify those with a high degree of intestinal damage, replacing the need for small bowel biopsies in some instances and suggesting that IgA autoantibodies can inform on tissue-specific biology^41^. Only one previous study reported elevated IgA in the serum of patients with DL versus patients with various other skin diseases^42^. The exact role of IgA in the development of DL remains unknown.

Interestingly, the majority of discoid lesions are negative for IgA deposits^43^, indicating that IgA deposition is not the primary driver of discoid lesions. However, IgA could still contribute secondarily to DL pathogenesis in parallel to other disease mechanisms. The finding that plasma IgA correlates with severity of DL is consistent with this hypothesis. To understand whether IgA is causally associated with DL, further work should be performed to investigate whether IgA deposits identify patients with more severe pathology than those without IgA deposits. Experimental depletion of IgA in patients with DL or animal models might also shed light on the causal role of IgA in DL.

By measuring a broad panel of inflammation-associated proteins in a cohort of patients with SLE with varying organ disease manifestations, we observed distinct inflammation proteins associated with different SLE organ disease manifestations. The markers identified in this study could be confirmed in larger population studies of patients with SLE to better assess the sensitivity and specificity of these markers for DL and LN. More information about the relationship between the identified proteins and LN and DL could also be elucidated by longitudinal studies. If the biomarkers we identified were shown to coincide with the onset of symptoms, there would be evidence that they are directly associated with the cause of the symptoms and reveal how changes in these blood proteins coincide with the onset of disease manifestations. If proven to be biomarkers of subclinical disease that can be identified prior to onset of symptoms, these proteins could be used to guide earlier clinical care in patients. Longitudinal analysis of these proteins in the context of conventional SLE immunosuppressants could yield insights into the mechanisms of action of these therapeutics. Furthermore, analysis of these proteins after therapeutic interventions that neutralise type I IFN signalling and SLE autoantibodies could shed further light into whether these proteins reflect inflammation independent to known SLE disease pathways. Additional studies with paired blood and tissue samples would also aid the understanding of how these serological features correlate with longitudinal changes in afflicted tissue.

These results suggest that different types of inflammation, many with high independence to autoantibodies, type I IFN, and composite disease activity metrics, impact lupus glomeruli and discoid lesions. Furthermore, the results indicate proteins from a single blood sample could be used to conveniently monitor multiple inflammatory pathways present in different organ systems without the need for tissue biopsies. Because SLE is characterised by such a wide range of pathologies across different organ systems, these accessible blood-based protein measurements could prove crucial to non-invasively monitoring disease activity and dissecting SLE pathology in future clinical studies.

## Patients and Methods

### Research participants, clinical data, and sample collection

Plasma samples were collected from 189 patients with SLE from the Hopkins Lupus Cohort. The cohort used in this study consisted of 18 patients with LN class III, 23 patients with LN class IV, 23 patients with LN class V, 25 patients with DL, 25 patients with lupus-associated thrombocytopenia, 50 patients with ACL, and 25 patients with secondary SS. LN classes were assessed using International Society of Nephrology (ISN)/Renal Pathology Society (RPS) criteria. Of these patients, 55% were African American, 40% Caucasian, and 3% Asian; 91% were female. All patients were examined by the same rheumatologist and met either the revised American College of Rheumatology 1982 or the Systemic Lupus International Collaborative Clinics classification criteria for SLE. Clinical data included comprehensive clinical laboratory tests and two disease activity measures, the Safety of Estrogens in Lupus Erythematosus–National Assessment Trial revision of the SLEDAI^44^ (SELENA-SLEDAI) and the LAI^45^ visual analogue scale. All patients were receiving standard of care at the time of sampling, with 71% receiving prednisone and 57% receiving hydroxychloroquine. Approximately 24% of patients were also receiving cytotoxic therapies, but no patients were being treated with biologics. Plasma samples from healthy donors were obtained anonymously from AstraZeneca/MedImmune employee volunteers through the MedImmune, LLC Research Specimen Collection Program in accordance with the guidelines of the Institutional Review Board of MedImmune, LLC, as previously described^46^. Exclusion criteria for this in-house collection programme included HIV infection, hepatitis B or C virus, human T-lymphotropic virus, or syphilis. Clinical characteristics for each SLE patient subset and healthy donors who consented to share demographic information are listed in Table 1. Distribution of disease activity scores for patients are listed in Supplemental Table 7.

The study was approved by the Johns Hopkins University School of Medicine Institutional Review Board. Methods were carried out in accordance with the relevant guidelines and regulations. All patients gave written informed consent (according to the Declaration of Helsinki).

### Plasma protein and autoantibody measurement

The prevalence of 100 plasma proteins was quantified using the RBM xMAP (Myriad RBM, Austin, TX, USA) from first thaw aliquots. Prevalence of IgG, IgM, and IgA autoantibodies targeting 94 SLE-related autoantigens were measured using an autoantigen microarray as previously described^47,48^. dsDNA-specific IgE was detected via ELISA as previously described^49^. IL-21 was measured via ELISA (cat. no. 433808; Biolegend, San Diego, CA, USA). All measurements are listed in Supplemental Table 2.

### Statistical analysis

All statistical analyses were performed in R. Pairwise group comparisons were performed using the non-parametric Mann–Whitney U test. AUC and 95% confidence intervals (CI) were produced with 2,000 stratified bootstrap replicates and reported as calculated using the pROC method. The non-parametric Spearman’s rank correlation test was used to measure associations between numeric variables. 95% CI for the Spearman’s rank correlation coefficient were reported using the Fisher’s z-transformation. FDR were calculated using the Benjamini and Hochberg procedure, and a cut-off of 0.10 was used to determine significance^50^.

Multivariate analysis was performed using logistic regression in R to measure the independent associations of different analytes with the dependent variable. *P* values and CI on statistics are reported using t-statistics calculated on the regression coefficients using the glm method available in R. Classifier performance was reported as the mean AUC, sensitivity, and specificity of the classifier on the test observations from 10 iterations of 5-fold cross validation. The analytes reported were not significantly associated with age, sex, race, or medication use in this cohort.

### Gene expression analysis

Gene expression measurements from microdissected glomeruli of patients with LN and healthy donors were used for analysis as previously published under GEO accession GSE32591^25^. Briefly, RNA was isolated using the RNAeasy Mini Kit (Qiagen, Valencia, CA, USA) and hybridised to Human Genome U133A gene chips (Affymetrix, Inc., Santa Clara, CA, USA)^25^. Gene expression values measured from PBMCs and whole blood samples of patients with LN and healthy donors were also used for analysis as previously published under GEO accessions GSE81622, GSE72798, and GSE65391^26–28^. In GSE81622, RNA from PBMCs of patients with LN was isolated using Trizol (Invitrogen Life Technologies, Carlsbad, CA, USA) and hybridised to Illumina Sentrix Expression Beadchips, Human HT-12v4 (Illumina, San Diego, CA, USA)^26^. In GSE72798, whole blood RNA was isolated from patients with LN using PAXgene Blood RNA tubes (Qiagen, Courtaboeuf, France), then hybridised to Human Genome U133 Plus 2.0 Arrays (Affymetrix, High Wycombe, UK)^27^. In GSE65391, whole blood RNA from paediatric patients with LN was isolated using the PerfectPure RNA Blood kit (5 PRIME Inc, Gaithersburg, MD, USA) and hybridised to Illumina Sentrix Expression Beadchips, Human HT-12v4 (Illumina, San Diego, CA, USA)^28^. Two sets of gene expression values from punch biopsies of discoid lesions and healthy donors were used for analysis as previously published under GEO accession GSE72535 and GSE52471^29,30^. In GEO72535, RNA was extracted using the RNeasy Lipid Tissue Mini Kit (Qiagen, Hilden, Germany) and hybridised to Illumina Sentrix Expression Beadchips, Human HT-12v4 (Illumina, San Diego, CA, USA)^29^. In GEO52471, RNA was extracted using the RNAeasy Mini Kit (Qiagen, Valencia, CA, USA) and hybridised to Human Genome U133A 2.0 gene chips (Affymetrix, Inc., Santa Clara, CA, USA).

## Supplementary information

Supplementary Information

## Data Availability

Data underlying the findings described in this manuscript may be obtained in accordance with AstraZeneca’s data sharing policy described at https://astrazenecagrouptrials.pharmacm.com/ST/Submission/Disclosure.
