# Supplementary material for: SLE Plasma Profiling Identifies Unique Signatures of Lupus Nephritis and Discoid Lupus
Source: Sci Rep. 2019 Oct 8;9:14433. doi: 10.1038/s41598-019-50231-y (PMC6783423; doi:10.1038/s41598-019-50231-y)
Supplement: Supplementary file 1 — Supplementary Information [file 41598_2019_50231_MOESM1_ESM.pdf]

SLE Plasma Profiling Identifies Unique Signatures of Lupus Nephritis and Discoid Lupus

Michael A. Smith, Jill Henault, Jodi L. Karnell, Melissa L. Parker, Jeffrey M. Riggs, Dominic Sinibaldi, Devon K. Taylor, Rachel Ettinger, Ethan P. Grant, Miguel A. Sanjuan, Roland Kolbeck, Michelle A. Petri, Kerry A. Casey.

Supplementary Tables

Supplemental Table 1. Analytes associated with proliferative nephritis

Variable	Comparison	AUC (95% CI)	N	Mann–Whitney *P* value	FDR
Anti-dsDNA IgG	LN III, IV vs LN V	0.83 (0.72, 0.92)	40, 22	< 0.001	1.98E-03
Anti-ssDNA IgG	LN III, IV vs LN V	0.81 (0.70, 0.91)	40, 22	< 0.001	3.50E-03
Anti-chromatin IgG	LN III, IV vs LN V	0.81 (0.69, 0.91)	40, 22	< 0.001	3.68E-03
Anti-nucleosome IgG	LN III, IV vs LN V	0.80 (0.68, 0.91)	40, 22	< 0.001	4.46E-03
Anti-dsRNA IgG	LN III, IV vs LN V	0.76 (0.63, 0.87)	40, 22	< 0.001	1.69E-02
Anti-histone H1 IgG	LN III, IV vs LN V	0.76 (0.63, 0.88)	40, 22	< 0.001	1.79E-02
Anti-histone H2B IgG	LN III, IV vs LN V	0.74 (0.61, 0.85)	40, 22	0.001	2.55E-02
Anti-histone H2A IgG	LN III, IV vs LN V	0.74 (0.60, 0.85)	40, 22	0.002	3.14E-02
Anti-nucleosome IgA	LN III, IV vs LN V	0.73 (0.59, 0.85)	40, 22	0.003	4.04E-02
Anti-chromatin IgM	LN III, IV vs LN V	0.72 (0.58, 0.84)	40, 22	0.004	5.07E-02
Anti-dsDNA IgE	LN III, IV vs LN V	0.64 (0.49, 0.77)	40, 22	0.07	2.9E-01
Anti-dsDNA IgG	LN III, IV vs no renal symptoms	0.76 (0.65, 0.86)	40, 22	< 0.001	5.17E-04
Anti-ssDNA IgG	LN III, IV vs no renal symptoms	0.71 (0.60, 0.80)	40, 22	< 0.001	6.57E-03
Anti-chromatin IgG	LN III, IV vs no renal symptoms	0.76 (0.65, 0.86)	40, 22	< 0.001	5.17E-04
Anti-nucleosome IgG	LN III, IV vs no renal symptoms	0.75 (0.63, 0.85)	40, 22	< 0.001	8.57E-04
Anti-dsRNA IgG	LN III, IV vs no renal symptoms	0.71 (0.60, 0.81)	40, 22	< 0.001	6.80E-03
Anti-histone H1 IgG	LN III, IV vs no renal symptoms	0.70 (0.59, 0.80)	40, 22	< 0.001	1.01E-02
Anti-histone H2B IgG	LN III, IV vs no renal symptoms	0.71 (0.60, 0.81)	40, 22	< 0.001	6.58E-03
Anti-histone H2A IgG	LN III, IV vs no renal symptoms	0.68 (0.57, 0.78)	40, 22	0.001	1.92E-02
Anti-nucleosome IgA	LN III, IV vs no renal symptoms	0.74 (0.64, 0.83)	40, 22	< 0.001	1.21E-03
Anti-chromatin IgM	LN III, IV vs no renal symptoms	0.65 (0.53, 0.75)	40, 22	0.008	7.50E-02
Anti-dsDNA IgE	LN III, IV vs no renal symptoms	0.70 (0.61, 0.79)	40, 22	< 0.001	4.87E-03

Anti-dsDNA = anti–double-stranded DNA; anti-ssDNA = Anti–single-stranded DNA; AUC = area under the curve; CI = confidence interval; FDR = false discovery rate; Ig = immunoglobulin; IV = intravenous; LN = lupus nephritis.

Supplemental Table 2. Analytes measured

Name	Type	Platform
Interleukin-21 (IL-21) (pg/ml)	Protein	ELISA
RF IgM (O.D.)	Antibody	ELISA
Ro IgG (O.D.)[a]	Antibody	ELISA
dsDNA IgG (ug/ml)[a]	Antibody	ELISA
IgM (ug/ml)	Antibody	ELISA
IgG (ug/ml)	Antibody	ELISA
dsDNA IgE	Antibody	ELISA
6Ckine (pg/mL)	Protein	RBM Custom xMAP
Adiponectin (ug/mL)	Protein	RBM Custom xMAP
Alpha-1 anti-trypsin (AAT) (mg/mL)	Protein	RBM Custom xMAP
Alpha-2-macroglobulin (A2Macro) (mg/mL)	Protein	RBM Custom xMAP
Angiopoietin-2 (ANG-2) (ng/mL)	Protein	RBM Custom xMAP
Apolipoprotein(a) (Lp(a)) (ug/mL)	Protein	RBM Custom xMAP
B-cell activating factor (BAFF) (pg/mL)	Protein	RBM Custom xMAP
B lymphocyte chemoattractant (BLC) (pg/mL)	Protein	RBM Custom xMAP
Beta-2-microglobulin (B2M) (ug/mL)	Protein	RBM Custom xMAP
Brain-derived neurotrophic factor (BDNF) (ng/mL)	Protein	RBM Custom xMAP
C-reactive protein (CRP) (ug/mL)	Protein	RBM Custom xMAP
Cancer antigen 15-3 (CA-15-3) (U/mL)	Protein	RBM Custom xMAP

Name	Type	Platform
CD 40 antigen (CD40) (ng/mL)	Protein	RBM Custom xMAP
CD40 ligand (CD40-L) (ng/mL)	Protein	RBM Custom xMAP
Complement C3 (C3) (mg/mL)	Protein	RBM Custom xMAP
Creatine kinase-MB (CK-MB) (ng/mL)	Protein	RBM Custom xMAP
Dickkopf-related protein 1 (DKK-1) (ng/mL)	Protein	RBM Custom xMAP
E-selectin (ng/mL)	Protein	RBM Custom xMAP
EN-RAGE (ng/mL)	Protein	RBM Custom xMAP
Eotaxin-1 (pg/mL)	Protein	RBM Custom xMAP
Eotaxin-2 (pg/mL)	Protein	RBM Custom xMAP
Eotaxin-3 (pg/mL)	Protein	RBM Custom xMAP
Epithelial-derived neutrophil-activating protein 78 (ENA-78) (ng/mL)	Protein	RBM Custom xMAP
Factor VII (ng/mL)	Protein	RBM Custom xMAP
Ferritin (FRTN) (ng/mL)	Protein	RBM Custom xMAP
Fibrinogen (mg/mL)	Protein	RBM Custom xMAP
Granulocyte colony-stimulating factor (G-CSF) (pg/mL)	Protein	RBM Custom xMAP
Granulocyte-macrophage colony-stimulating factor (GM-CSF) (pg/mL)	Protein	RBM Custom xMAP
Haptoglobin (mg/mL)	Protein	RBM Custom xMAP
Immunoglobulin A (IgA) (mg/mL)	Protein	RBM Custom xMAP
Immunoglobulin E (IgE) (U/mL)	Protein	RBM Custom xMAP
Immunoglobulin M (IgM) (mg/mL)	Protein	RBM Custom xMAP

Name	Type	Platform
Insulin-like growth factor-binding protein 2 (IGFBP-2) (ng/mL)	Protein	RBM Custom xMAP
Intercellular adhesion molecule 1 (ICAM-1) (ng/mL)	Protein	RBM Custom xMAP
Interferon alpha (IFN-alpha) (ng/mL)	Protein	RBM Custom xMAP
Interferon gamma (IFN-gamma) (pg/mL)	Protein	RBM Custom xMAP
Interferon-gamma-induced protein 10 (IP-10) (pg/mL)	Protein	RBM Custom xMAP
Interferon-inducible T-cell alpha chemoattractant (ITAC) (pg/mL)	Protein	RBM Custom xMAP
Interleukin-1 alpha (IL-1 alpha) (ng/mL)	Protein	RBM Custom xMAP
Interleukin-1 beta (IL-1 beta) (pg/mL)	Protein	RBM Custom xMAP
Interleukin-1 receptor antagonist (IL-1ra) (pg/mL)	Protein	RBM Custom xMAP
Interleukin-2 (IL-2) (pg/mL)	Protein	RBM Custom xMAP
Interleukin-2 receptor alpha (IL-2 receptor alpha) (pg/mL)	Protein	RBM Custom xMAP
Interleukin-3 (IL-3) (ng/mL)	Protein	RBM Custom xMAP
Interleukin-4 (IL-4) (pg/mL)	Protein	RBM Custom xMAP
Interleukin-5 (IL-5) (pg/mL)	Protein	RBM Custom xMAP
Interleukin-6 (IL-6) (pg/mL)	Protein	RBM Custom xMAP
Interleukin-6 receptor (IL-6r) (ng/mL)	Protein	RBM Custom xMAP
Interleukin-7 (IL-7) (pg/mL)	Protein	RBM Custom xMAP
Interleukin-8 (IL-8) (pg/mL)	Protein	RBM Custom xMAP
Interleukin-10 (IL-10) (pg/mL)	Protein	RBM Custom xMAP
Interleukin-12 subunit p40 (IL-12p40) (ng/mL)	Protein	RBM Custom xMAP

Name	Type	Platform
Interleukin-12 subunit p70 (IL-12p70) (pg/mL)	Protein	RBM Custom xMAP
Interleukin-13 (IL-13) (pg/mL)	Protein	RBM Custom xMAP
Interleukin-15 (IL-15) (ng/mL)	Protein	RBM Custom xMAP
Interleukin-16 (IL-16) (pg/mL)	Protein	RBM Custom xMAP
Interleukin-17 (IL-17) (pg/mL)	Protein	RBM Custom xMAP
Interleukin-18 (IL-18) (pg/mL)	Protein	RBM Custom xMAP
Interleukin-22 (IL-22) (ng/mL)	Protein	RBM Custom xMAP
Interleukin-23 (IL-23) (ng/mL)	Protein	RBM Custom xMAP
Interleukin-31 (IL-31) (ng/mL)	Protein	RBM Custom xMAP
Latency-associated peptide of transforming growth factor beta-1 (LAP TGF-b1) (ng/mL)	Protein	RBM Custom xMAP
Macrophage-derived chemokine (MDC) (pg/mL)	Protein	RBM Custom xMAP
Macrophage inflammatory protein-1 alpha (MIP-1 alpha) (pg/mL)	Protein	RBM Custom xMAP
Macrophage inflammatory protein-1 beta (MIP-1 beta) (pg/mL)	Protein	RBM Custom xMAP
Macrophage inflammatory protein-3 alpha (MIP-3 alpha) (pg/mL)	Protein	RBM Custom xMAP
Macrophage inflammatory protein-3 beta (MIP-3 beta) (pg/mL)	Protein	RBM Custom xMAP
Macrophage migration inhibitory factor (MIF) (ng/mL)	Protein	RBM Custom xMAP
Matrix metalloproteinase-3 (MMP-3) (ng/mL)	Protein	RBM Custom xMAP
Matrix metalloproteinase-9 (MMP-9) (ng/mL)	Protein	RBM Custom xMAP
Monocyte chemotactic protein-1 (MCP-1) (pg/mL)	Protein	RBM Custom xMAP
Monocyte chemotactic protein-2 (MCP-2) (pg/mL)	Protein	RBM Custom xMAP

Name	Type	Platform
Monocyte chemotactic protein-4 (MCP-4) (pg/mL)	Protein	RBM Custom xMAP
Monokine induced by gamma interferon (MIG) (pg/mL)	Protein	RBM Custom xMAP
Myeloid progenitor inhibitory factor-1 (MPIF-1) (ng/mL)	Protein	RBM Custom xMAP
Myeloperoxidase (MPO) (ng/mL)	Protein	RBM Custom xMAP
Myoglobin (ng/mL)	Protein	RBM Custom xMAP
Osteoprotegerin (OPG) (pM)	Protein	RBM Custom xMAP
Plasminogen activator inhibitor-1 (PAI-1) (ng/mL)	Protein	RBM Custom xMAP
Prostate-specific antigen, free (PSA-f) (ng/mL)	Protein	RBM Custom xMAP
Serum amyloid P component (SAP) (ug/mL)	Protein	RBM Custom xMAP
Sex hormone-binding globulin (SHBG) (mmol/L)	Protein	RBM Custom xMAP
Stem cell factor (SCF) (pg/mL)	Protein	RBM Custom xMAP
Stromal cell-derived factor-1 (SDF-1) (pg/mL)	Protein	RBM Custom xMAP
T-cell-specific protein RANTES (ng/mL)	Protein	RBM Custom xMAP
Thrombospondin-1 (ng/mL)	Protein	RBM Custom xMAP
Thymus and activation-regulated chemokine (TARC) (ng/mL)	Protein	RBM Custom xMAP
Thyroxine-binding globulin (TBG) (ug/mL)	Protein	RBM Custom xMAP
Tissue inhibitor of metalloproteinases-1 (TIMP-1) (ng/mL)	Protein	RBM Custom xMAP
Tumour necrosis factor alpha (TNF-alpha) (pg/mL)	Protein	RBM Custom xMAP
Tumour necrosis factor beta (TNF-beta) (pg/mL)	Protein	RBM Custom xMAP
Tumour necrosis factor ligand superfamily member 12 (Tweak) (ng/mL)	Protein	RBM Custom xMAP

Name	Type	Platform
Tumour necrosis factor ligand superfamily member 13 (APRIL) (ng/mL)	Protein	RBM Custom xMAP
Tumour necrosis factor receptor I (TNF RI) (pg/mL)	Protein	RBM Custom xMAP
Tumour necrosis factor receptor 2 (TNFR2) (ng/mL)	Protein	RBM Custom xMAP
Vascular cell adhesion molecule-1 (VCAM-1) (ng/mL)	Protein	RBM Custom xMAP
Vascular endothelial growth factor (VEGF) (pg/mL)	Protein	RBM Custom xMAP
Vitamin D binding protein (VDBP) (ug/mL)	Protein	RBM Custom xMAP
Vitronectin (ug/mL)	Protein	RBM Custom xMAP
von Willebrand factor (vWF) (ug/mL)	Protein	RBM Custom xMAP
Anti-aggrecan IgA, IgG, IgM	Antibody	UTSW Autoantigen Microarray
Anti-alpha-actinin IgA, IgG, IgM	Antibody	UTSW Autoantigen Microarray
Anti-amyloid IgA, IgG, IgM	Antibody	UTSW Autoantigen Microarray
Anti-beta 2-glycoprotein I IgA, IgG, IgM	Antibody	UTSW Autoantigen Microarray
Anti-beta 2-microglobulin IgA, IgG, IgM	Antibody	UTSW Autoantigen Microarray
Anti-BPI IgA, IgG, IgM	Antibody	UTSW Autoantigen Microarray
Anti-C1q IgA, IgG, IgM	Antibody	UTSW Autoantigen Microarray
Anti-cardolipin IgA, IgG, IgM	Antibody	UTSW Autoantigen Microarray
Anti-CENP-A IgA, IgG, IgM	Antibody	UTSW Autoantigen Microarray
Anti-CENP-B IgA, IgG, IgM	Antibody	UTSW Autoantigen Microarray
Anti-chondroitin sulfate C IgA, IgG, IgM	Antibody	UTSW Autoantigen Microarray
Anti-chromatin IgA, IgG, IgM	Antibody	UTSW Autoantigen Microarray

Name	Type	Platform
Anti-collagen I IgA, IgG, IgM	Antibody	UTSW Autoantigen Microarray
Anti-collagen II IgA, IgG, IgM	Antibody	UTSW Autoantigen Microarray
Anti-collagen III IgA, IgG, IgM	Antibody	UTSW Autoantigen Microarray
Anti-collagen IV IgA, IgG, IgM	Antibody	UTSW Autoantigen Microarray
Anti-collagen V IgA, IgG, IgM	Antibody	UTSW Autoantigen Microarray
Anti-collagen VI IgA, IgG, IgM	Antibody	UTSW Autoantigen Microarray
Anti-cytochrome C IgA, IgG, IgM	Antibody	UTSW Autoantigen Microarray
Anti-decorin-bovine IgA, IgG, IgM	Antibody	UTSW Autoantigen Microarray
Anti-DGPS IgA, IgG, IgM	Antibody	UTSW Autoantigen Microarray
Anti-ds RNA IgA, IgG, IgM	Antibody	UTSW Autoantigen Microarray
Anti-dsDNA IgA, IgG, IgM	Antibody	UTSW Autoantigen Microarray
Anti-elastin IgA, IgG, IgM	Antibody	UTSW Autoantigen Microarray
Anti-entactin EDTA IgA, IgG, IgM	Antibody	UTSW Autoantigen Microarray
Anti-fibrinogen IV IgA, IgG, IgM	Antibody	UTSW Autoantigen Microarray
Anti-fibrinogen S IgA, IgG, IgM	Antibody	UTSW Autoantigen Microarray
Anti-fibronectin IgA, IgG, IgM	Antibody	UTSW Autoantigen Microarray
Anti-GBM-dissociated IgA, IgG, IgM	Antibody	UTSW Autoantigen Microarray
Anti-gliadin-IgG IgA, IgG, IgM	Antibody	UTSW Autoantigen Microarray
Anti-glycated albumin-human IgA, IgG, IgM	Antibody	UTSW Autoantigen Microarray
Anti-GP2 IgA, IgG, IgM	Antibody	UTSW Autoantigen Microarray

Name	Type	Platform
Anti-gP210 IgA, IgG, IgM	Antibody	UTSW Autoantigen Microarray
Anti-haemocyanin IgA, IgG, IgM	Antibody	UTSW Autoantigen Microarray
Anti-heparan HSPG IgA, IgG, IgM	Antibody	UTSW Autoantigen Microarray
Anti-heparin IgA, IgG, IgM	Antibody	UTSW Autoantigen Microarray
Anti-heparan Sulfate IgA, IgG, IgM	Antibody	UTSW Autoantigen Microarray
Anti-histone H3 IgA, IgG, IgM	Antibody	UTSW Autoantigen Microarray
Anti-histone H4 IgA, IgG, IgM	Antibody	UTSW Autoantigen Microarray
Anti-histone H1 IgA, IgG, IgM	Antibody	UTSW Autoantigen Microarray
Anti-histone H2A IgA, IgG, IgM	Antibody	UTSW Autoantigen Microarray
Anti-histone H2B IgA, IgG, IgM	Antibody	UTSW Autoantigen Microarray
Anti-histone-total IgA, IgG, IgM	Antibody	UTSW Autoantigen Microarray
Anti-intrinsic factor IgA, IgG, IgM	Antibody	UTSW Autoantigen Microarray
Anti-Jo-1 IgA, IgG, IgM	Antibody	UTSW Autoantigen Microarray
Anti-KU-P70/P80 IgA, IgG, IgM	Antibody	UTSW Autoantigen Microarray
Anti-La/SSB IgA, IgG, IgM	Antibody	UTSW Autoantigen Microarray
Anti-laminin IgA, IgG, IgM	Antibody	UTSW Autoantigen Microarray
Anti-LC1 IgA, IgG, IgM	Antibody	UTSW Autoantigen Microarray
Anti-LKM1 IgA, IgG, IgM	Antibody	UTSW Autoantigen Microarray
Anti-M2 antigen IgA, IgG, IgM	Antibody	UTSW Autoantigen Microarray
Anti-MAG-myelin-associated glycoprotein-FC IgA, IgG, IgM	Antibody	UTSW Autoantigen Microarray

Name	Type	Platform
Anti-matrigel IgA, IgG, IgM	Antibody	UTSW Autoantigen Microarray
Anti-MBP-myelin basic protein IgA, IgG, IgM	Antibody	UTSW Autoantigen Microarray
Anti-Mi-2 IgA, IgG, IgM	Antibody	UTSW Autoantigen Microarray
Anti-mitochondrial antigen IgA, IgG, IgM	Antibody	UTSW Autoantigen Microarray
Anti-MPO IgA, IgG, IgM	Antibody	UTSW Autoantigen Microarray
Anti-myosin IgA, IgG, IgM	Antibody	UTSW Autoantigen Microarray
Anti-nucleolin IgA, IgG, IgM	Antibody	UTSW Autoantigen Microarray
Anti-nucleosome antigen IgA, IgG, IgM	Antibody	UTSW Autoantigen Microarray
Anti-Nup62 IgA, IgG, IgM	Antibody	UTSW Autoantigen Microarray
Anti-PCNA IgA, IgG, IgM	Antibody	UTSW Autoantigen Microarray
Anti-peroxiredoxin 1 IgA, IgG, IgM	Antibody	UTSW Autoantigen Microarray
Anti-phosphatidylinositol IgA, IgG, IgM	Antibody	UTSW Autoantigen Microarray
Anti-PL-12 IgA, IgG, IgM	Antibody	UTSW Autoantigen Microarray
Anti-PL-7 IgA, IgG, IgM	Antibody	UTSW Autoantigen Microarray
Anti-PM/Scl-100 IgA, IgG, IgM	Antibody	UTSW Autoantigen Microarray
Anti-PM/Scl-75 IgA, IgG, IgM	Antibody	UTSW Autoantigen Microarray
Anti-PR3 IgA, IgG, IgM	Antibody	UTSW Autoantigen Microarray
Anti-proteoglycan IgA, IgG, IgM	Antibody	UTSW Autoantigen Microarray
Anti-prothrombin protein IgA, IgG, IgM	Antibody	UTSW Autoantigen Microarray
Anti-ribo phosphoprotein P1 IgA, IgG, IgM	Antibody	UTSW Autoantigen Microarray

Name	Type	Platform
Anti-ribo phosphoprotein P2 IgA, IgG, IgM	Antibody	UTSW Autoantigen Microarray
Anti-ribo phosphoprotein P0 IgA, IgG, IgM	Antibody	UTSW Autoantigen Microarray
Anti-Ro/SSA-52KDa IgA, IgG, IgM	Antibody	UTSW Autoantigen Microarray
Anti-Ro/SSA-60KDa IgA, IgG, IgM	Antibody	UTSW Autoantigen Microarray
Anti-Scl-70 IgA, IgG, IgM	Antibody	UTSW Autoantigen Microarray
Anti-Sm IgA, IgG, IgM	Antibody	UTSW Autoantigen Microarray
Anti-Sm/RNP IgA, IgG, IgM	Antibody	UTSW Autoantigen Microarray
Anti-SmD IgA, IgG, IgM	Antibody	UTSW Autoantigen Microarray
Anti-SP100 IgA, IgG, IgM	Antibody	UTSW Autoantigen Microarray
Anti-sphingomyelin IgA, IgG, IgM	Antibody	UTSW Autoantigen Microarray
Anti-SRP54 IgA, IgG, IgM	Antibody	UTSW Autoantigen Microarray
Anti-ssDNA IgA, IgG, IgM	Antibody	UTSW Autoantigen Microarray
Anti-thyroglobulin IgA, IgG, IgM	Antibody	UTSW Autoantigen Microarray
Anti-topoisomerase I IgA, IgG, IgM	Antibody	UTSW Autoantigen Microarray
Anti-TPO IgA, IgG, IgM	Antibody	UTSW Autoantigen Microarray
Anti-TTG IgA, IgG, IgM	Antibody	UTSW Autoantigen Microarray
Anti-U1-snRNP-68 IgA, IgG, IgM	Antibody	UTSW Autoantigen Microarray
Anti-U1-snRNP-A IgA, IgG, IgM	Antibody	UTSW Autoantigen Microarray
Anti-U1-snRNP-BB IgA, IgG, IgM	Antibody	UTSW Autoantigen Microarray
Anti-U1-snRNP-C IgA, IgG, IgM	Antibody	UTSW Autoantigen Microarray

Name	Type	Platform
Anti-vimentin IgA, IgG, IgM	Antibody	UTSW Autoantigen Microarray
Anti-vitronectin IgA, IgG, IgM	Antibody	UTSW Autoantigen Microarray

[a] Denotes antibodies measured by ELISA that were also included in the UTSW Autoantigen Microarray.

Supplemental Table 3. Analytes associated with glomerulonephritis (LN III, LN IV, LN V)

Variable	Comparison	AUC (95% CI)	N	Mann–Whitney *P* value*	FDR
CD 40 antigen (CD40)	All LN vs no renal symptoms	0.70 (0.61, 0.79)	96, 64	1.39E-05	4.45E-04
Fibrinogen	All LN vs no renal symptoms	0.71 (0.62, 0.79)	96, 64	7.19E-06	2.34E-04
Interleukin-1 beta (IL-1 beta)	All LN vs no renal symptoms	0.74 (0.66, 0.81)	96, 64	4.20E-07	4.14E-05
Interleukin-1 receptor antagonist (IL-1ra)	All LN vs no renal symptoms	0.66 (0.58, 0.75)	96, 64	4.72E-04	5.39E-03
Interleukin-22 (IL-22)	All LN vs no renal symptoms	0.71 (0.62, 0.78)	96, 64	6.23E-06	2.32E-04
Stem cell factor (SCF)	All LN vs no renal symptoms	0.70 (0.62, 0.78)	96, 64	1.43E-05	3.96E-04
Tissue inhibitor of metalloproteinases 1 (TIMP-1)	All LN vs no renal symptoms	0.75 (0.67, 0.82)	96, 64	8.49E-08	1.69E-05
Tumour necrosis factor receptor 2 (TNFR2)	All LN vs no renal symptoms	0.77 (0.70, 0.84)	96, 64	4.31E-09	2.21E-06
Vascular cell adhesion molecule-1 (VCAM-1)	All LN vs no renal symptoms	0.74 (0.66, 0.81)	96, 64	4.41E-07	4.84E-05
Vascular endothelial growth factor (VEGF)	All LN vs no renal symptoms	0.72 (0.64, 0.81)	96, 64	1.79E-06	1.15E-04
CD 40 antigen (CD40)	LN III vs no renal symptoms	0.75 (0.6, 0.88)	96, 18	7.84E-04	--
Fibrinogen	LN III vs no renal symptoms	0.73 (0.56, 0.88)	96, 18	1.65E-03	--
Interleukin-1 beta (IL-1 beta)	LN III vs no renal symptoms	0.67 (0.52, 0.81)	96, 18	2.20E-02	--
Interleukin-1 receptor antagonist (IL-1ra)	LN III vs no renal symptoms	0.68 (0.52, 0.82)	96, 18	1.75E-02	--
Interleukin-22 (IL-22)	LN III vs no renal symptoms	0.75 (0.6, 0.87)	96, 18	6.13E-04	--
Stem cell factor (SCF)	LN III vs no renal symptoms	0.67 (0.50, 0.82)	96, 18	2.62E-02	--
Tissue inhibitor of metalloproteinases 1 (TIMP-1)	LN III vs no renal symptoms	0.77 (0.64, 0.88)	96, 18	3.10E-04	--
Tumour necrosis factor receptor 2 (TNFR2)	LN III vs no renal symptoms	0.84 (0.73, 0.93)	96, 18	5.15E-06	--

Variable	Comparison	AUC (95% CI)	N	Mann–Whitney *P* value*	FDR
Vascular cell adhesion molecule-1 (VCAM-1)	LN III vs no renal symptoms	0.74 (0.62, 0.85)	96, 18	1.13E-03	--
Vascular endothelial growth factor (VEGF)	LN III vs no renal symptoms	0.67 (0.51, 0.82)	96, 18	2.54E-02	--
CD 40 antigen (CD40)	LN IV vs no renal symptoms	0.71 (0.57, 0.84)	96, 23	1.42E-03	--
Fibrinogen	LN IV vs no renal symptoms	0.67 (0.54, 0.80)	96, 23	1.02E-02	--
Interleukin-1 beta (IL-1 beta)	LN IV vs no renal symptoms	0.74 (0.62, 0.83)	96, 23	4.61E-04	--
Interleukin-1 receptor antagonist (IL-1ra)	LN IV vs no renal symptoms	0.65 (0.53, 0.76)	96, 23	2.58E-02	--
Interleukin-22 (IL-22)	LN IV vs no renal symptoms	0.71 (0.58, 0.83)	96, 23	1.40E-03	--
Stem cell factor (SCF)	LN IV vs no renal symptoms	0.77 (0.67, 0.86)	96, 23	5.65E-05	--
Tissue inhibitor of metalloproteinases 1 (TIMP-1)	LN IV vs no renal symptoms	0.77 (0.65, 0.87)	96, 23	7.13E-05	--
Tumour necrosis factor receptor 2 (TNFR2)	LN IV vs no renal symptoms	0.84 (0.74, 0.92)	96, 23	6.41E-07	--
Vascular cell adhesion molecule-1 (VCAM-1)	LN IV vs no renal symptoms	0.76 (0.66, 0.85)	96, 23	1.29E-04	--
Vascular endothelial growth factor (VEGF)	LN IV vs no renal symptoms	0.73 (0.62, 0.84)	96, 23	5.07E-04	--
CD 40 antigen (CD40)	LN V vs no renal symptoms	0.65 (0.51, 0.79)	96, 23	2.19E-02	--
Fibrinogen	LN V vs no renal symptoms	0.73 (0.61, 0.84)	96, 23	7.46E-04	--
Interleukin-1 beta (IL-1 beta)	LN V vs no renal symptoms	0.79 (0.68, 0.88)	96, 23	1.70E-05	--
Interleukin-1 receptor antagonist (IL-1ra)	LN V vs no renal symptoms	0.67 (0.55, 0.78)	96, 23	1.37E-02	--
Interleukin-22 (IL-22)	LN V vs no renal symptoms	0.67 (0.53, 0.80)	96, 23	7.91E-03	--
Stem cell factor (SCF)	LN V vs no renal symptoms	0.66 (0.53, 0.79)	96, 23	1.52E-02	--
Tissue inhibitor of metalloproteinases 1 (TIMP-1)	LN V vs no renal symptoms	0.72 (0.59, 0.84)	96, 23	1.16E-03	--

Variable	Comparison	AUC (95% CI)	N	Mann–Whitney *P* value*	FDR
Tumour necrosis factor receptor 2 (TNFR2)	LN V vs no renal symptoms	0.66 (0.53, 0.79)	96, 23	1.58E-02	--
Vascular cell adhesion molecule-1 (VCAM-1)	LN V vs no renal symptoms	0.71 (0.59, 0.81)	96, 23	1.92E-03	--
Vascular endothelial growth factor (VEGF)	LN V vs no renal symptoms	0.76 (0.63, 0.87)	96, 23	1.39E-04	--

*Mann–Whitney adjusted P value < 0.10, elevated in each subset P value < 0.05.
AUC = area under the curve; CI = confidence interval; FDR = false discovery rate; IV = intravenous; LN = lupus nephritis.

Supplemental Table 4. Correlation between proteins associated with glomerulonephritis and renal LAI and renal SLEDAI

Variable	Comparison	Spearman's R (95% CI)	N	*P* value
CD 40 antigen (CD40)	Renal LAI	0.34 (0.48, 0.19)	186	2.74E-06
Fibrinogen	Renal LAI	0.32 (0.47, 0.18)	186	6.62E-06
Interleukin-1 beta (IL-1 beta)	Renal LAI	0.32 (0.47, 0.18)	186	7.97E-06
Interleukin-1 receptor antagonist (IL-1ra)	Renal LAI	0.33 (0.48, 0.19)	186	4.14E-06
Interleukin-22 (IL-22)	Renal LAI	0.35 (0.49, 0.20)	186	1.09E-06
Stem cell factor (SCF)	Renal LAI	0.34 (0.49, 0.20)	186	1.80E-06
Tissue inhibitor of metalloproteinases 1 (TIMP-1)	Renal LAI	0.46 (0.61, 0.32)	186	3.57E-11
Tumour necrosis factor receptor 2 (TNFR2)	Renal LAI	0.39 (0.53, 0.24)	186	3.86E-08
Vascular cell adhesion molecule-1 (VCAM-1)	Renal LAI	0.31 (0.45, 0.16)	186	1.77E-05
Vascular endothelial growth factor (VEGF)	Renal LAI	0.38 (0.52, 0.23)	186	1.09E-07
CD 40 antigen (CD40)	Renal SLEDAI	0.29 (0.43, 0.14)	186	6.00E-05
Fibrinogen	Renal SLEDAI	0.28 (0.43, 0.14)	186	1.06E-04
Interleukin-1 beta (IL-1 beta)	Renal SLEDAI	0.27 (0.41, 0.12)	186	2.42E-04
Interleukin-1 receptor antagonist (IL-1ra)	Renal SLEDAI	0.28 (0.42, 0.13)	186	1.18E-04
Interleukin-22 (IL-22)	Renal SLEDAI	0.19 (0.33, 0.04)	186	1.00E-02
Stem cell factor (SCF)	Renal SLEDAI	0.27 (0.41, 0.12)	186	2.33E-04
Tissue inhibitor of metalloproteinases 1 (TIMP-1)	Renal SLEDAI	0.33 (0.47, 0.18)	186	4.49E-06
Tumour necrosis factor receptor 2 (TNFR2)	Renal SLEDAI	0.30 (0.45, 0.16)	186	2.75E-05
Vascular cell adhesion molecule-1 (VCAM-1)	Renal SLEDAI	0.20 (0.35, 0.06)	186	5.49E-03
Vascular endothelial growth factor (VEGF)	Renal SLEDAI	0.24 (0.38, 0.09)	186	1.15E-03

LAI = Lupus Activity Index; SLEDAI = Systemic Lupus Erythematosus Disease Activity Index.

Supplemental Table 5. Logistic regression model measuring independent associations between plasma protein and presence of renal symptoms or lupus nephritis*

	Coefficient (95% CI)	*P* value
Intercept	-4.30 (-5.9, -2.9)	< 0.001
Tumour necrosis factor receptor 2 (TNFR2)	0.14 (0.076, 0.22)	< 0.001
Interleukin-1 beta (IL-1 beta)	0.24 (0.032, 0.41)	0.0038
Fibrinogen	0.50 (0.14, 0.87)	0.0074

*Mean 10 iterations 5-fold cross validation (AUC = 0.80, Sensitivity = 0.63, Specificity = 0.89).
AUC = area under the curve.

Supplemental Table 6. Analytes associated with discoid lupus

Variable	Comparison	AUC (95% CI)	N	Mann–Whitney *P* value*	FDR
Interleukin-21 (IL-21)	Discoid vs no cutaneous symptoms	0.28 (0.17, 0.41)	77, 25	1.27E-03	4.00E-02
Interleukin-23 (IL-23)	Discoid vs no cutaneous symptoms	0.67 (0.55, 0.79)	85, 25	8.51E-03	9.57E-02
Serum amyloid P-component (SAP)	Discoid vs no cutaneous symptoms	0.68 (0.57, 0.79)	85, 25	5.55E-03	8.78E-02
IgA - beta 2-microglobulin	Discoid vs no cutaneous symptoms	0.73 (0.61, 0.83)	78, 23	1.05E-03	3.47E-02
IgA - KU-P70/P80	Discoid vs no cutaneous symptoms	0.71 (0.57, 0.83)	78, 23	2.92E-03	7.05E-02
IgA - LC1	Discoid vs no cutaneous symptoms	0.69 (0.58, 0.8)	78, 23	4.77E-03	8.56E-02
IgA - TTG	Discoid vs no cutaneous symptoms	0.73 (0.61, 0.84)	78, 23	9.67E-04	3.74E-02
IgG - fibrinogen IV	Discoid vs no cutaneous symptoms	0.68 (0.55, 0.8)	78, 23	9.00E-03	9.58E-02
IgG - histone H4	Discoid vs no cutaneous symptoms	0.74 (0.64, 0.84)	78, 23	3.36E-04	2.11E-02
IgG - peroxiredoxin 1	Discoid vs no cutaneous symptoms	0.78 (0.68, 0.86)	78, 23	6.01E-05	7.54E-03
IgG - U1-snRNP-C	Discoid vs no cutaneous symptoms	0.68 (0.57, 0.8)	78, 23	7.62E-03	9.58E-02
Interleukin-21 (IL-21)	Discoid vs acute cutaneous lupus	0.34 (0.21, 0.48)	46, 25	2.66E-02	--
Interleukin-23 (IL-23)	Discoid vs acute cutaneous lupus	0.71 (0.57, 0.83)	49, 25	3.83E-03	--
Serum amyloid P-component (SAP)	Discoid vs acute cutaneous lupus	0.73 (0.6, 0.84)	49, 25	1.18E-03	--
IgA - beta 2-microglobulin	Discoid vs acute cutaneous lupus	0.65 (0.51, 0.79)	48, 23	3.73E-02	--
IgA - KU-P70/P80	Discoid vs acute cutaneous lupus	0.72 (0.59, 0.85)	48, 23	2.36E-03	--
IgA - LC1	Discoid vs acute cutaneous lupus	0.7 (0.57, 0.81)	48, 23	8.10E-03	--
IgA - TTG	Discoid vs acute cutaneous lupus	0.71 (0.59, 0.83)	48, 23	3.96E-03	--
IgG - fibrinogen IV	Discoid vs acute cutaneous lupus	0.6 (0.46, 0.74)	48, 23	1.63E-01	--
IgG - histone H4	Discoid vs acute cutaneous lupus	0.83 (0.73, 0.91)	48, 23	6.67E-06	--
IgG - peroxiredoxin 1	Discoid vs acute cutaneous lupus	0.78 (0.66, 0.88)	48, 23	1.30E-04	--
IgG - U1-snRNP-C	Discoid vs acute cutaneous lupus	0.7 (0.58, 0.82)	48, 23	6.26E-03	--

*Mann–Whitney adjusted *P* value < 0.10.
AUC = area under the curve; FDR = false discovery rate.

Supplemental Table 7. Distribution of disease activity scores in cohort

	All SLE (n = 189)	Discoid (n = 25)	LN III (n = 18)	LN IV (n = 23)	LN V (n = 23)	Thrombocytopenia (n = 25)	ACL (n = 50)	Sjögren's (n = 25)
**SLEDAI**								
Seizure (%)	0	0	0	0	0	0	0	0
Psychosis (%)	0	0	0	0	0	0	0	0
Organic brain syndrome (%)	0	0	0	0	0	0	0	0
Visual disturbance (%)	2	4	0	0	0	0	0	8
Cranial nerve disorder (%)	1	0	0	4	0	0	0	0
Lupus headache (%)	0	0	0	0	0	0	0	0
Cerebrovascular accident (%)								
Vasculitis (%)	3	8	0	4	0	0	4	0
Urinary Casts (%)	1	0	6	0	0	0	0	0
Haematuria (%)	6	8	22	0	4	0	6	4
Proteinuria (%)	11	16	6	13	13	13	6	12
Pyuria (%)	3	0	17	0	9	0	0	0
Arthritis (%)	9	0	6	4	0	13	14	16
Myositis (%)	1	0	0	0	0	0	2	0
Low complement (%)	45	56	50	61	43	48	32	40
Increased DNA binding (%)	41	40	56	70	26	43	30	36
Rash (%)	34	16	0	9	9	17	100	8
Alopecia (%)	9	4	28	4	13	0	12	4

	All SLE (n = 189)	Discoid (n = 25)	LN III (n = 18)	LN IV (n = 23)	LN V (n = 23)	Thrombocytopenia (n = 25)	ACL (n = 50)	Sjögren's (n = 25)
Mucosal ulcers (%)	3	8	0	0	0	0	4	4
Pleurisy (%)	3	0	6	4	4	4	0	4
Pericarditis (%)	0	0	0	0	0	0	0	0
Thrombocytopenia (%)	6	0	0	4	0	25	4	12
Leukopenia (%)	9	16	0	13	0	17	6	8
Fever (%)	0	0	0	0	0	0	0	0
**LAI**								
Median fatigue (1Q, 3Q)	0(0,0)	0(0,0)	0(0,0)	0(0,0)	0(0,0)	0(0,0)	0(0,0)	0(0,0)
Median rash (1Q, 3Q)	0(0,1)	0(0,1)	0(0,0)	0(0,0)	0(0,0)	0(0,0)	1(0,1)	0(0,0)
Median joints (1Q, 3Q)	0(0,0)	0(0,0)	0(0,0)	0(0,0)	0(0,0)	0(0,0)	0(0,1)	0(0,0)
Median serous (1Q, 3Q)	0(0,0)	0(0,0)	0(0,0)	0(0,0)	0(0,0)	0(0,0)	0(0,0)	0(0,0)
Median neurology (1Q, 3Q)	0(0,0)	0(0,0)	0(0,0)	0(0,0)	0(0,0)	0(0,0)	0(0,0)	0(0,0)
Median renal (1Q, 3Q)	0(0,1)	0(0,0)	0(0,1)	0(0,1)	1(0,2)	0(0,0)	0(0,0)	0(0,0)
Median pulmonary (1Q, 3Q)	0(0,0)	0(0,0)	0(0,0)	0(0,0)	0(0,0)	0(0,0)	0(0,0)	0(0,0)
Median haematology (1Q, 3Q)	0(0,0)	0(0,0)	0(0,0)	0(0,0)	0(0,0)	0(0,0)	0(0,0)	0(0,0)

SLEDAI = Systemic Lupus Erythematosus Disease Activity Index; LAI = Lupus Activity Index; HD = healthy donors; LN = lupus nephritis; ACL = acute cutaneous lupus; 1Q = first quartile; 3Q = third quartile.
